# Ferumoxytole-enhanced 4D MR angiography with retrospectively defined temporal resolution

**DOI:** 10.1186/1532-429X-18-S1-P363

**Published:** 2016-01-27

**Authors:** Ziwu Zhou, Fei Han, Yu Gao, Takegawa Yoshida, Peng Hu, J Paul Finn

**Affiliations:** Radiological Sciences, University of California, Los Angeles, Los Angeles, CA USA

## Background

In cardiovascular MRI applications, high spatial resolution is preferable for anatomical evaluation of vascular structures and high temporal resolution is desired in cardiac functional assessment. As a result, current CMR protocol usually includes repeated scans of the same anatomy with different resolution settings. To address this issue, we propose a technique that allows for retrospective trade-off between temporal and spatial resolution based on a single image acquisition. The proposed technique is applied on Ferumoxytol-enhanced 4D angiography application so that images with high temporal and high spatial resolution are available from separated reconstructions.

## Methods

**Figure 1:**
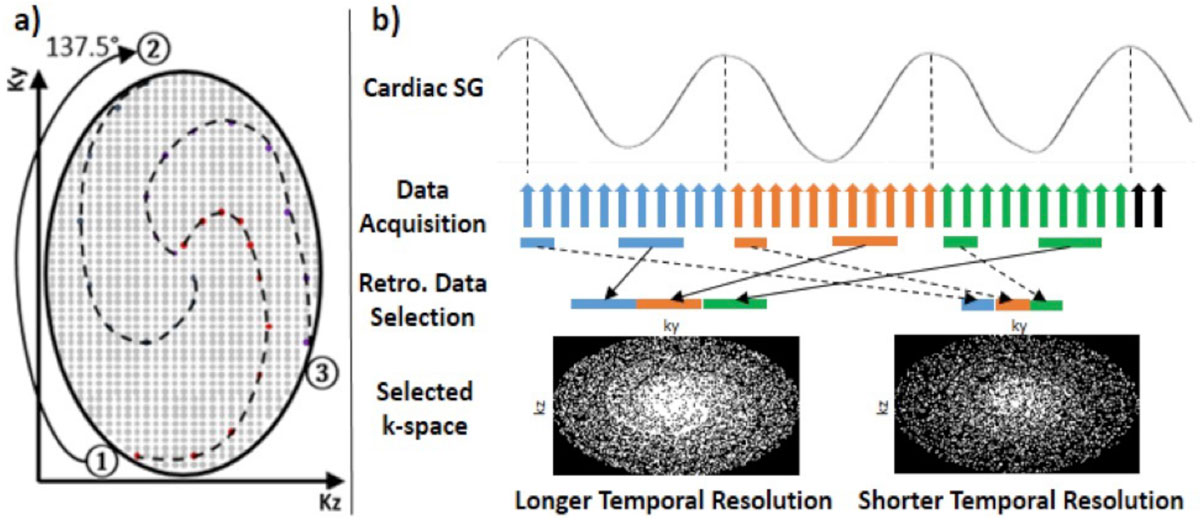
**(a) Modified k-space under-sampling pattern with rotating quasi-spiral arms**. (b) Acquired data were retrospectively selected and binned into different cardiac phases based on detected self-gating (SG) triggers. The acquired dataset can be binned into different cardiac phases using different configurations on reconstruction window width and position.

A 3D Cartesian sequence was modified using ROtating Cartesian K-space (ROCK) reordering methods where ky-kz plane is sampled in a quasi-spiral pattern with consecutive interleaves rotated by golden angle (Figure 1a). Each quasi-spiral interleave starts from the k-space center-line, from which respiratory and cardiac motion can be estimated using self-gating techniques. After the acquisition, the data is retrospectively gated and binned using derived self-gated signals (Figure 1b). The k-space bins are then reconstructed using L1-ESPIRiT with spatial wavelet regularization. For the highly under-sampled k-space bins (i.e. high temporal resolution), temporal total variation is used in the reconstruction as an additional regularization.

9 clinical indicated pediatric patients with congenital heart disease were included in this study. General anesthesia was performed with controlled mechanical ventilation. Scan was performed on a Siemens 3.0T scanner with the following parameters: TE/TR = 0.9/2.9 ms, FA = 25, FOV = 500 × 300 × 150 mm3, 0.8-1 mm isotropic resolution without interpolation, TA~6 min. In this study, each dataset was binned using two settings: 6 cardiac phases@80 ms and 18 phases@25 ms. Image reconstruction was performed in our CPU and GPU accelerated inline image reconstruction system.

## Results

**Figure 2:**
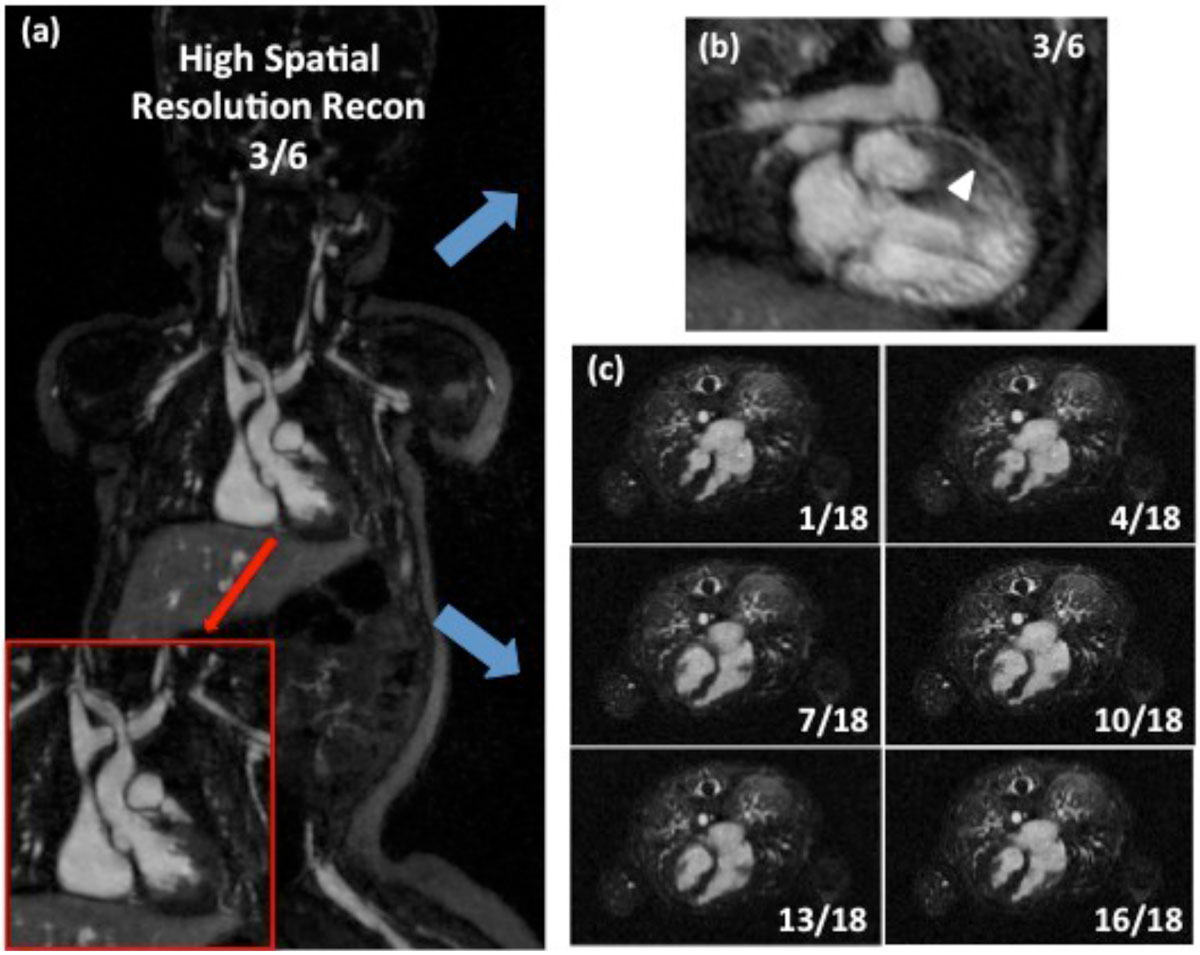
**Images acquired from a 3-month-old female with congenital heart disease**. Vascular and cardiac structures (including major coronary artery) are well defined in the image reconstructed with high spatial resolution in 6 cardiac phases (a,b). The images reconstructed in high temporal resolution have 18 cardiac phase (c), which is suitable for LV functional assessment. The total acquisition time is 4 min with respiratory motion gating.

Figure 2a shows the images from the 6-cardiac-phase-reconstruction on a 3-month-old female. Major vessels and cardiac structures are clearly defined in these high spatial resolution images. The left anterior descending (LAD) artery (arrow) is visualized on a reformatted 2D image in the third cardiac phase shown in Figure 2b. Figure 2c shows 6 of the 18 cardiac phase reconstructions based on the same dataset where the blood-myocardium boarders are clearly defined. The high contrast between myocardium and blood-pool could potentially facilitate an automatic ventricular segmentation for fast and accurate left ventricle functional measurements.

## Conclusions

The proposed ROCK sampling pattern and data binning strategy enables retrospectively defined temporal resolution. Our initial results suggest that simultaneous anatomical and functional assessment is possible based on two separate reconstructions of a single 4D scan, although further validation on subjective image quality scores and quantitative LV functional measurements is warranted.

